# Scorpions and life-history strategies: from evolutionary dynamics toward the scorpionism problem

**DOI:** 10.1186/s40409-018-0160-0

**Published:** 2018-08-22

**Authors:** Wilson R. Lourenço

**Affiliations:** Muséum national d’Histoire naturelle, Sorbonne Universités, Institut de Systématique, Evolution, Biodiversité (ISYEB), UMR7205-CNRS, MNHN, UPMC, EPHE, CP 53, 57 rue Cuvier, 75005 Paris, France

**Keywords:** Scorpion, Reproductive strategies, Embryonic, Postembryonic development

## Abstract

This work aims to contribute to the general information on scorpion reproductive patterns in general including species that can be noxious to humans. Scorpions are unusual among terrestrial arthropods in several of their life-history traits since in many aspects their reproductive strategies are more similar to those of superior vertebrates than to those of arthropods in general. This communication focuses mainly on the aspects concerning embryonic and post-embryonic developments since these are quite peculiar in scorpions and can be directly connected to the scorpionism problem. As in previous similar contributions, the content of this communication is addressed mainly to non-specialists whose research embraces scorpions in several fields such as venom toxins and public health. A precise knowledge of reproductive strategies presented by several scorpion groups and, in particular, those of dangerous species may prove to be a useful tool in the interpretation of results dealing with scorpionism, and also lead to a better treatment of the problems caused by infamous scorpions.

## Background

In a series of previous publications addressed to the readers of the *Journal of Venomous Animals and Toxins including Tropical Diseases*, I attempted to provide some general information about scorpions and scorpionism, broadly addressed to non-specialists whose research embraces scorpions in several fields such as venom toxins and public health [1–6]. Most of the information previously supplied concerned historical aspects of scorpion studies but also several questions on their taxonomy, evolution and geographic distribution [1–6].

It is obvious, however that the scorpionism problem is not only associated with the evolution of toxins in some groups (families and genera) of scorpions, but also has close connections with their life-history strategies. For example, the ecology of a given population can directly influence its presence in nearby human habitations [2]. Similarly, reproductive strategies are globally associated with the dynamics of each scorpion population. General aspects of scorpion ecology and ecophysiology remain incompletely studied but will not be the subject of the present communication. Contrarily, many reproductive aspects of scorpions are presently known and attest to the strong particularities in their mode of reproduction [7]. In many aspects, scorpion reproductive strategies are more similar to those of superior vertebrates than to those of arthropods in general. In this communication, I will focus on the aspects concerning embryonic and post-embryonic developments since these aspects are peculiar to scorpions and have direct connection with scorpionism problems. Furthermore, I will avoid discussing behavioral aspects that are of less interest in relation to the problem of scorpionism. Much information has been published for more than a century on the development of the scorpions, but it is usually available in specialized literature, and is so scattered that it becomes unavailable for non-experts on the subject. Previous syntheses are already old [7, 8]; consequently a new presentation should be welcome to a broad audience.

The synthesis presented in this communication is mainly based on my personal research on scorpion reproductive biology performed during more than 40 years. It must, however be considered incomplete, since our global knowledge on scorpions’ biology still presents numerous gaps. For some extremely poorly studied groups no data are presently available. Nevertheless, the proposal of a more accessible synopsis appears to be valid in relation to the non-expert readers of the journal.

## General presentation

**Fig. 1:**
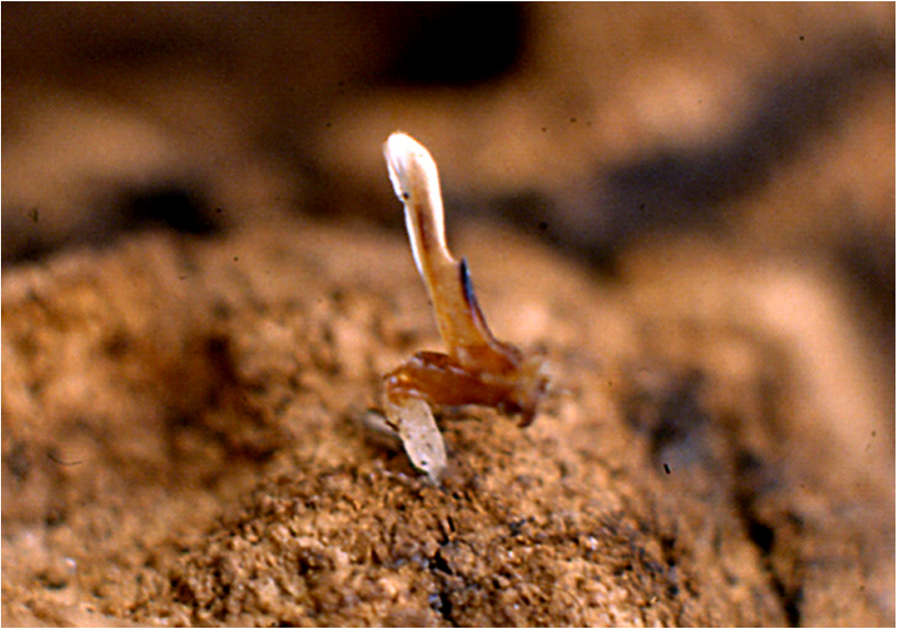
Deposited spermatophore of *Opisthacanthus cayaporum* (Brazilian hormurid) in a piece of bark, after the parturition process

As already outlined in previous publications [7, 8] scorpions are unusual among terrestrial arthropods in several traits of their life history. They show ritualized and complex courtship with fertilization by means of a spermatophore (Fig. 1), undergo, without any exception, viviparous embryonic developments, which can last from a few months to more than two years, and show some remarkable maternal care [9–11] followed in several species by an important degree of social behavior [12]. Their post-embryonic developments can be extraordinarily long, lasting from 7 to 130 months [7, 8].

On account of the unusual traits of their life history, many aspects of the reproductive biology of scorpions were poorly understood by pioneering authors, such as the classical “promenade à deux” described by Maccary [13] and Fabre [14]. Only by the middle of the 1950s, several researchers discovered, independently, that sperm transfer was accomplished by means of a spermatophore. The first reports were those by Angermann [15] and Alexander [16].

Nevertheless, long before the middle of the twentieth Century, detailed studies of scorpion embryology were developed by authors such as Laurie [17–20], which were followed by those of Pavlovsky [21, 22] and Pflugfelder [23]. Naturally, all these authors worked mainly through the techniques of comparative morphology based only on preserved specimens. Observations on living specimens started to take place by the 1950–1960s [24–26]. After these pioneering original contributions, much less attention was paid to embryology and only isolated publications provided additional information [27–35].

The first contribution on the post-embryonic development of scorpions was the publication by Schultze [36]. Beginning in the mid-1950s, several accounts of various aspects of the reproductive biology, in some cases of the entire post-embryonic development of scorpions, have been published. These were mainly by biologists such as Alexander [16, 37, 38], Auber [39, 40], Matthiesen [25, 26], Maury [41, 42], Shulov and Amitai [43], Shulov et al. [44], Varela [45] and Williams [46]. More recently other biological cycles of scorpions were studied [11, 47–53]. Several of these works dealt with groups never before observed. Naturally, these citations are certainly not exhaustive; a more complete list of references can be found in Polis and Sissom [8] and Lourenço [7].

The mid-1970s saw a renewal of interest in the reproductive biology of scorpions and particularly in their post-embryonic development. Research on this subject was multiplied during the 1980s and continued throughout the 1990s and 2000s. Interestingly, most of the authors of this work were taxonomists who, in addition to obtaining biological information, were investigating the ontogenetic variability of the characters used in taxonomy (see Lourenço [7] for references). The contributions by Polis and Farley [54, 55] represented an interesting exception since these authors attempted for the first time to explain reproductive traits in the context of evolutionary ecology. However, in more recent years disengagement toward biological studies has been observed since almost all the attention is given to phylogenetic and molecular studies.

A great disparity is evident from the known biological data in relation to the used methodology and the quality of the observations made. In many cases, the information reported may be speculative or even fallacious. I will not, however, discuss these aspects here.

## Why may biological data be important in the understanding of the scorpionism problem?

Interpretation of the ecology and geographical distribution patterns of scorpions cannot be achieved without a precise knowledge of the reproductive biology of these animals. Their capacity for a passive dispersion (normally transport by human agency) and possible re-adaptation to different and/or largely modified environments is totally dependent on their reproductive strategies. For this reason, scorpion species are generally divided into two categories: (i) equilibrium species, and (ii) opportunistic species. The reproduction models observed in these two categories are generally in accordance with their ecological requirements. Equilibrium species are totally dependent on stable and predictable environments, whereas opportunistic species have weak ecological requirements and are able to colonize highly modified and unpredictable environments. I will emphasize in the following sections two aspects of scorpion reproduction that are factors related to their definition as equilibrium or opportunistic species, and to their embryonic and post-embryonic developments. For more details on other aspects of scorpion reproduction, the reader can refer to Polis and Sissom [8] and Lourenço [7], where these aspects are presented in full detail.

## Embryonic development

**Fig. 2:**
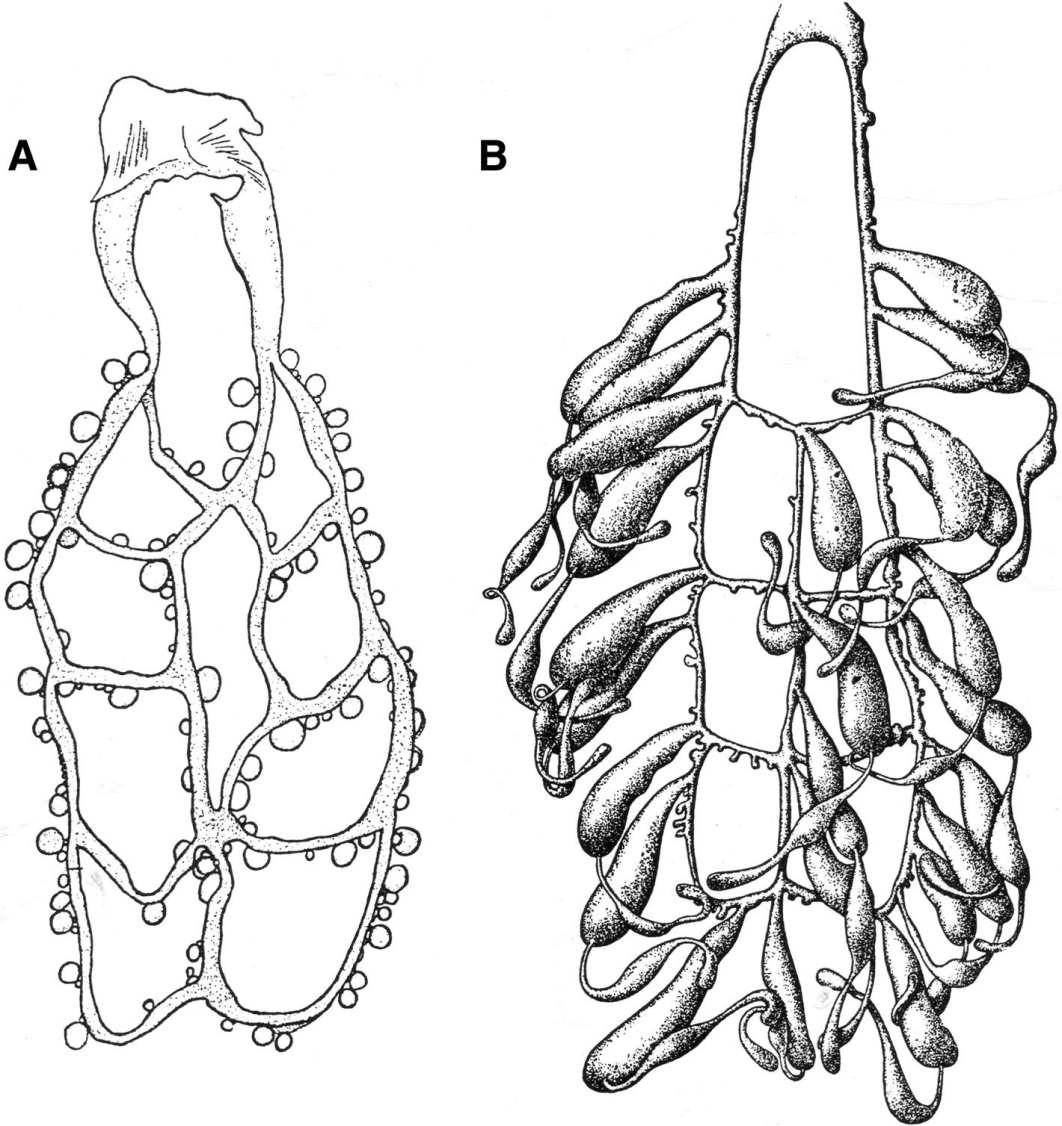
Ovariuterus in dorsal aspect of (**a**) *Rhopalurus rochai* (apoikogenic species) and (**b**) *Scorpio maurus* (katoikogenic species). Modified as described by Matthiersen [29] and Millot and Vachon [70]

**Fig. 3:**
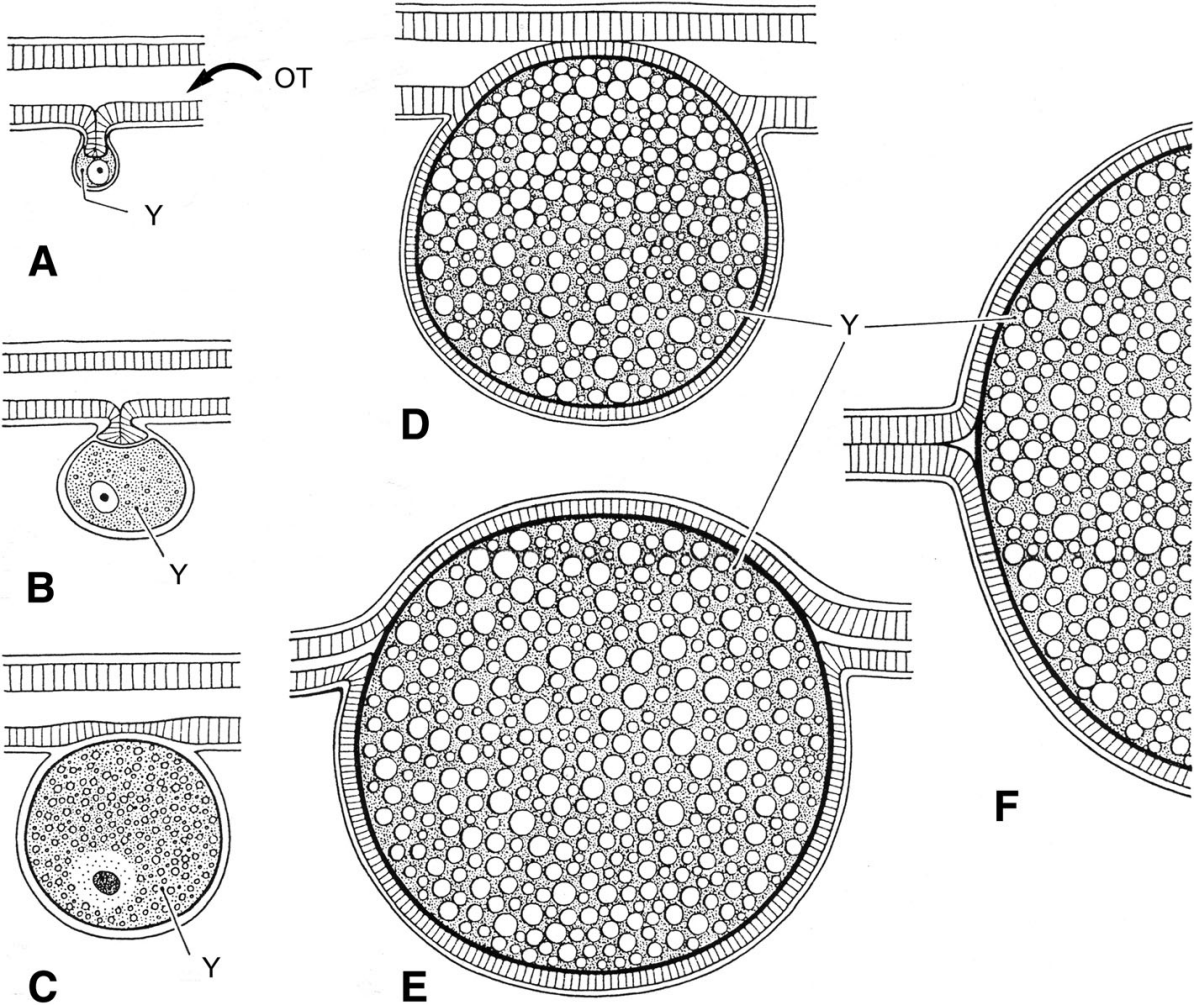
Schematic embryonic development of a buthid scorpion. (**a-c**) Early development of ovum outside ovariuterine tubule (OT). (**d-f**) Migration to the tubule and development yolk-producing cells of germinative epithelium (Y)

**Fig. 4:**
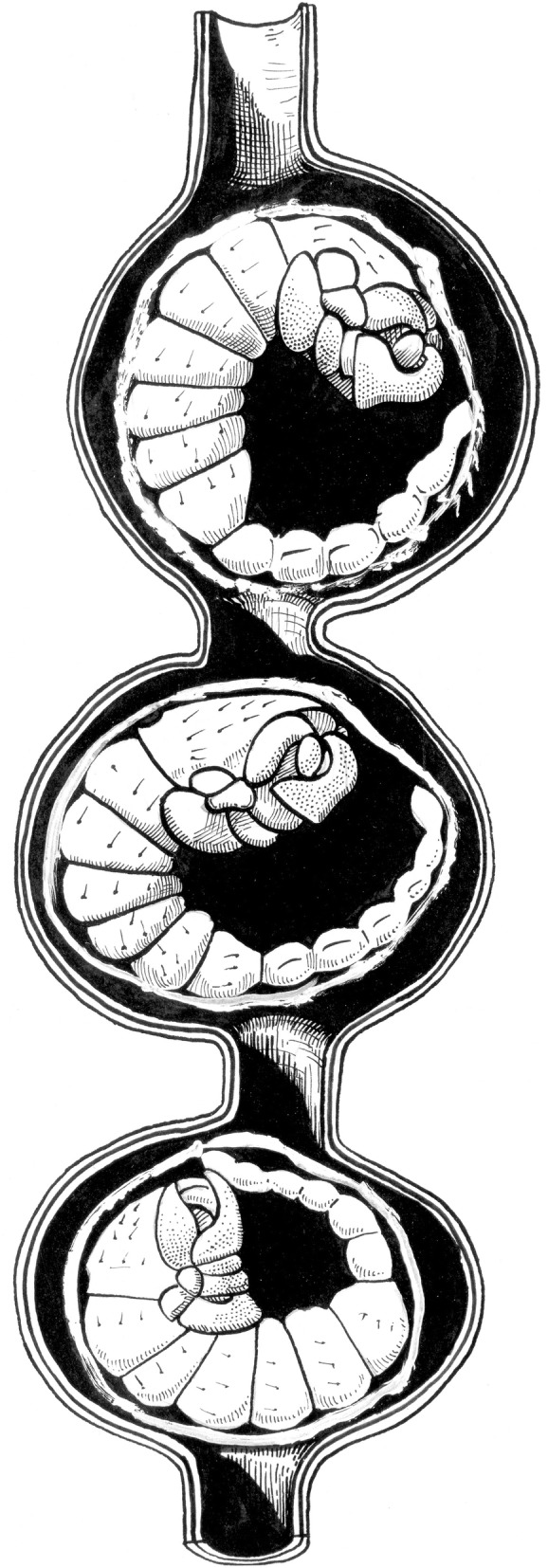
Schema showing well developed embryos of a buthid scorpion (apoikogenic species), already entirely migrated to the tubule

**Fig. 5:**
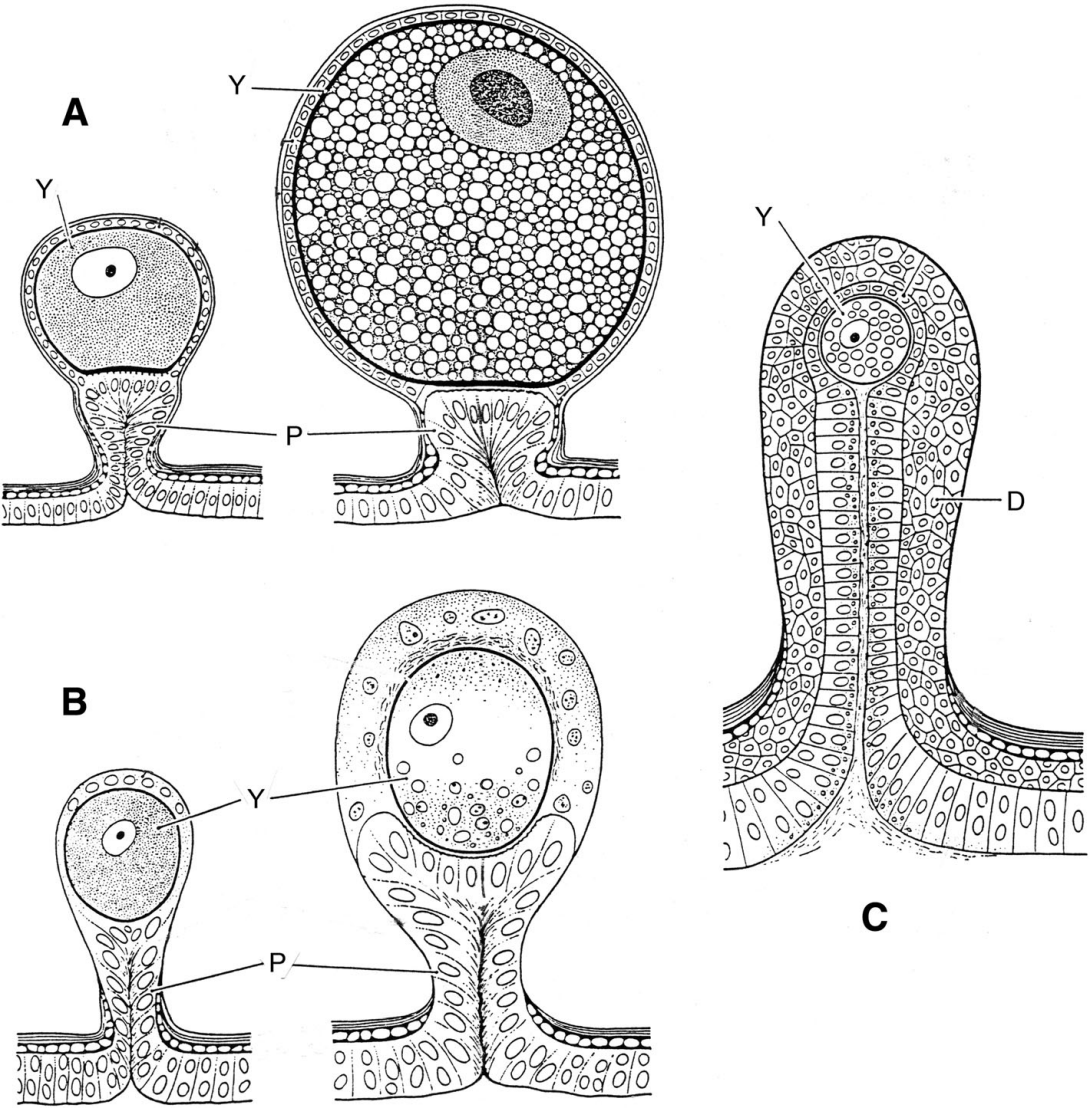
Schematic embryonic development in (**a**) euscorpiid, (**b**) vaejovid and (**c**) hormurid scorpions. Yolk-producing cells of germinative epithelium (Y). P: peduncle; D: diverticulum

**Fig. 6:**
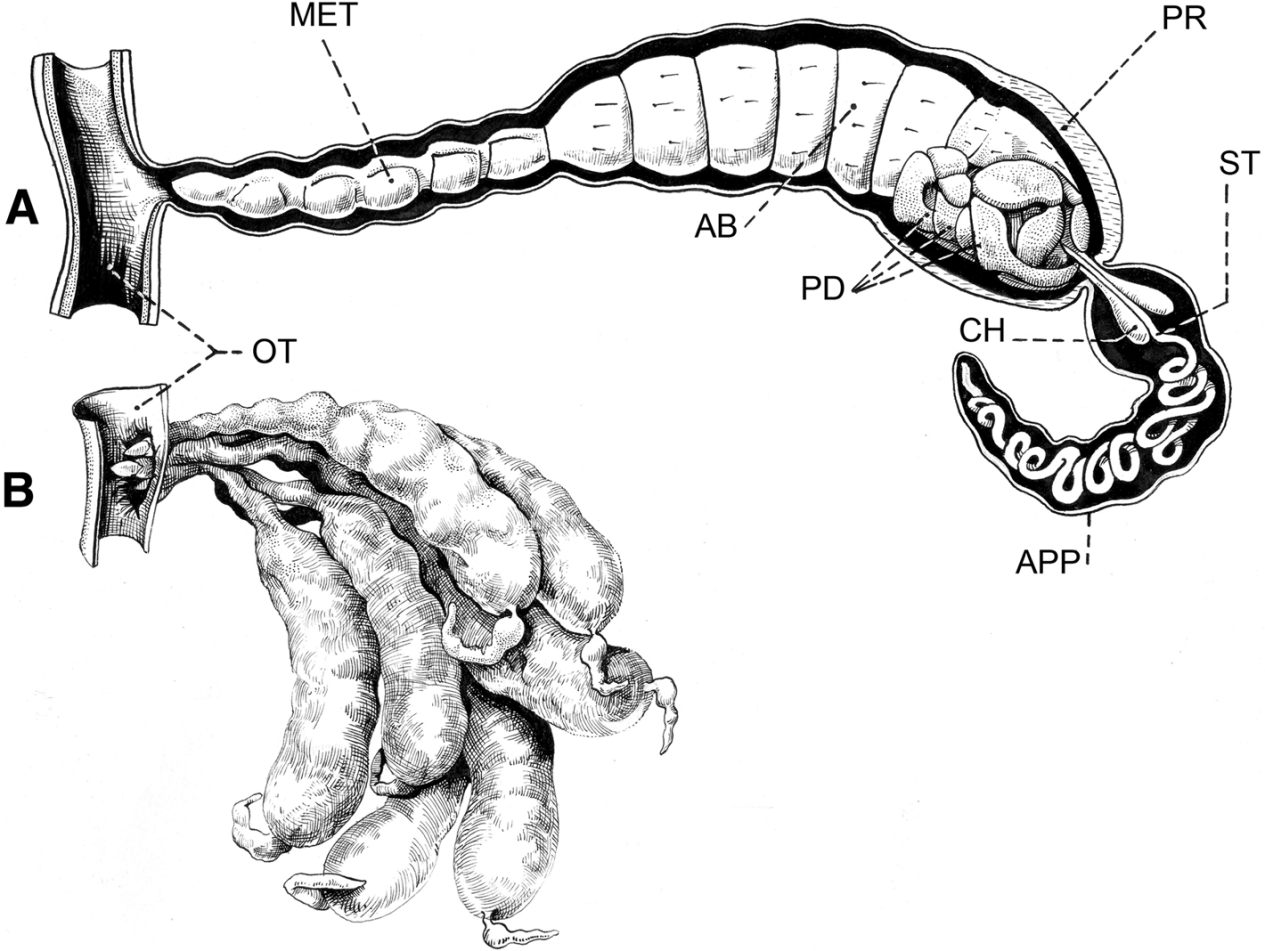
Schemas showing well developed embryos of the hormurid *Chiromachus ochropus* (katoikogenic species) from the Seychelles Islands. **a** Diverticulum with one embryo. **b** Several diverticula. OT: ovariuterine tubule; APP: appendix; CH: chelicerae; ST: spiral tubule; PR: prosoma; PD: pedipalps; AB: abdomen; MET: metasoma

It is now accepted by the majority of authors that viviparity is the only model of embryonic reproduction in all scorpion species [32–35], although until recently some authors still suggested that certain species could be ovoviviparous. At the end of the nineteenth Century, two classical models of embryonic development were proposed by Laurie [19] and defined as apoikogenic and katoikogenic. These models are still retained by most authors (see Polis and Sissom [8] and Lourenço [7] for references). Laurie’s [19] model suggests a dichotomy in the embryonic development of scorpions; the first type is development without the presence of diverticula, called apoikogenic – which in Greek means *from outside of the home* – and the second one, a development with the presence of diverticula called katoikogenic – meaning *at home* in Greek (Figs. 2, 3, 4, 5 and 6).

**Fig. 7:**
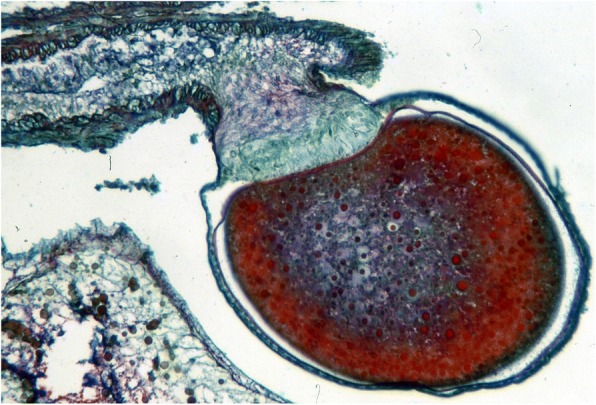
Histological section showing the development of the apoikogenic buthid *Centruroides barbudensis*

**Fig. 8:**
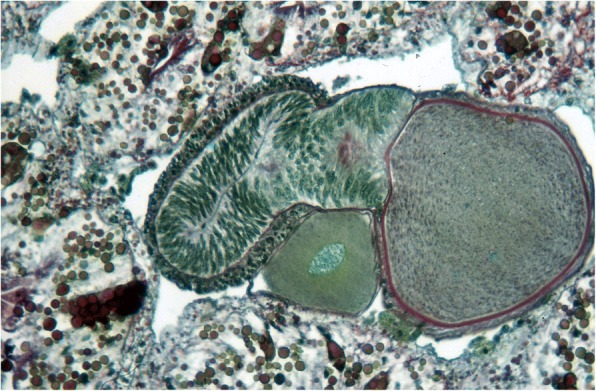
Histological section showing the development of the apoikogenic euscorpiid *Euscorpius flavicaudis*

**Fig. 9:**
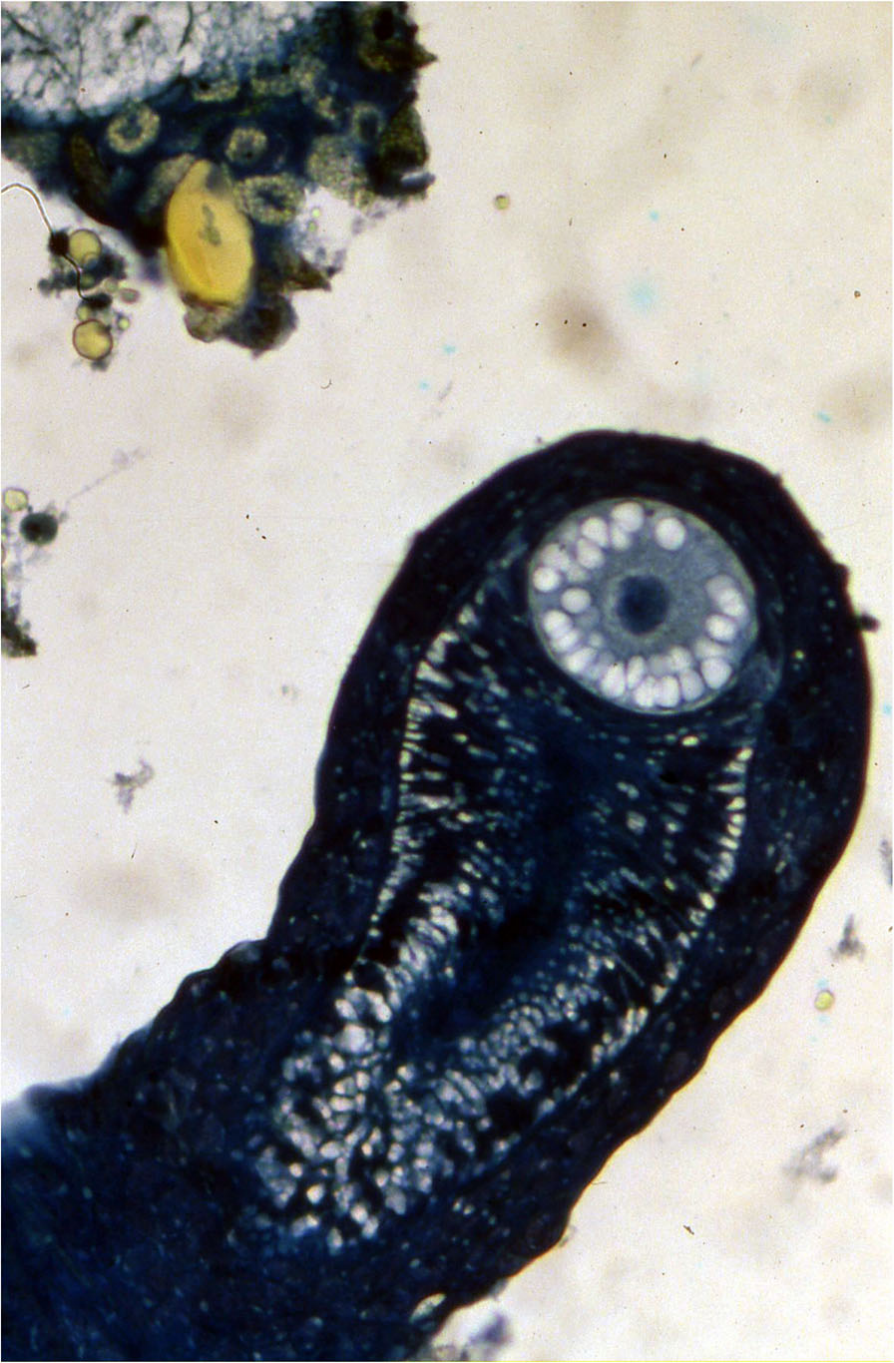
Histological section showing the development of the katoikogenic hormurid *Opisthacanthus asper*

Ninety years after Laurie [19], Lourenço et al. [33, 34] proposed a new concept of the embryological development of scorpions which partially modified the classical apoikogenic and katoikogenic models. According to this model, viviparity occurs in all scorpions studied, as previously suggested by Francke [32]. The approach is based on tissue modification of the ovaries and differentiation associated with the formation of the ovarian follicles. Following this amended model, a number of familial lineages can be arranged along a gradient of increasing complexity of viviparous development. Trophic exchanges that occur between the mother and the embryos range from the simplest at the apoikogenic base to the most complex type at the katoikogenic apex (Figs. 7, 8 and 9).

The most complex gradients of embryonic development with well-developed diverticula are exhibited by the best known scorpion families including the Scorpionidae, Diplocentridae, Hormuridae, Hadogenidae, Urodacidae, and most certainly the Hemiscorpiidae and Heteroscorpionidae (Fig. 9). In other families – such as the Buthidae, Bothriuridae, Chactidae, Euscorpiidae, Scorpiopidae, Superstitioniidae, Vaejovidae, Iuridae and Chaerilidae – the gradients range from simple to moderately complex (Figs. 7 and 8). No data are available for poorly known families, such as the Microcharmidae, Troglotayosicidae and Pseudochactidae.

According to the model of embryonic development and also in association with the species’ lineage, the duration times of this development can show some huge variations ranging from 2.5 months for some buthid species to 24 months for some species of the family Hormuridae. Nearly complete tables are available in Polis and Sissom [8] and Lourenço [7].

**Fig. 10:**
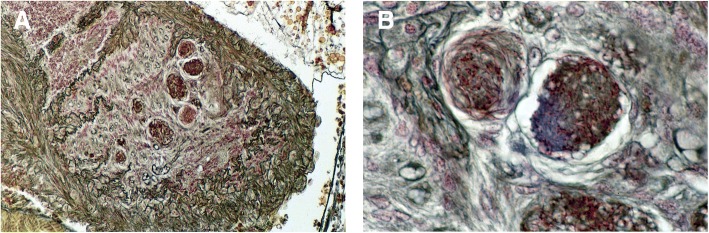
Storage of spermatozoa in glandular tissue of the buthid *Centruroides barbudensis*. (**a**) Part of the proximal glandular region of the ovarian tube containing piles of spermatozoa. (**b**) Details of sperm masses surrounded by glandular cells

Another important aspect of the embryonic process, presented by some scorpion species, is the capacity of females to produce multiple broods after a single insemination. Notably, this aspect is distinct from iteroparity, which is defined as repeated reproduction during the lifetime of a female. In fact, iteroparity seems to be present in all studied scorpions. The precise mechanism associated with this production of multiple broods remained unsolved for many decades but was finally clarified during the studies conducted by Kovoor et al. [35]. These authors demonstrated the existence of a unique process of storage of spermatozoa that are embedded in glandular tissue in the genital tract of the female (Fig. 10). This process was confirmed for several species belonging to the family Buthidae and in particular to some genera such as *Centruroides* and *Tityus*. Naturally, the process was also established in species belonging to other genera such as *Isometrus*, following a common process of evolution within the buthid lineage.

After a single insemination, females of species belonging to these genera are able to give birth to as many as five broods when isolated in laboratory conditions. *Isometrus maculatus* represents a particular case since the number of broods can reach seven in this species [56]. These observations have a bearing on the interpretation of the reproductive strategies of scorpions. Storage of spermatozoa can greatly increase the reproductive potential of some species and is most significant given that two of the buthid genera concerned (*Centruroides* and *Tityus*) contain species of medical importance that are responsible for thousands of incidents in which human beings are stung and not infrequently killed.

**Fig. 11:**
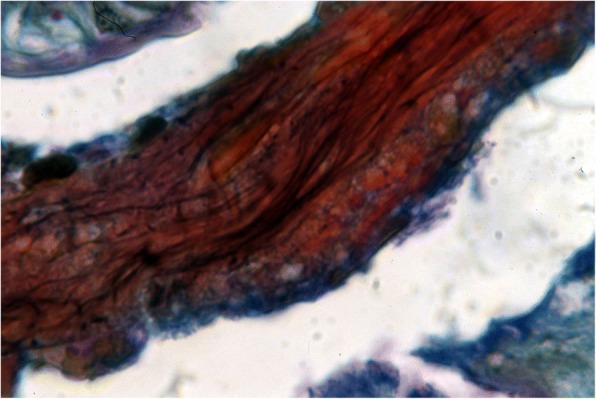
Modality of sperm conservation in the diplocentrid scorpion, *Didymocentrus lesueurii*, showing a heterogeneous mass in the genital atrium and the proximal region of the ovarian tube with bundles of spermatozoa inside the heterogeneous mass

Histological studies of an important number of non-buthid scorpions confirmed the absence of any effective sperm storage mechanism. In a few diplocentrid and hormurid species, only a much simpler modality of temporary conservation of spermatozoa in the genital atrium and the proximal region of the ovarian tube has been observed (Figs. 11).

## Brood size

**Fig. 12:**
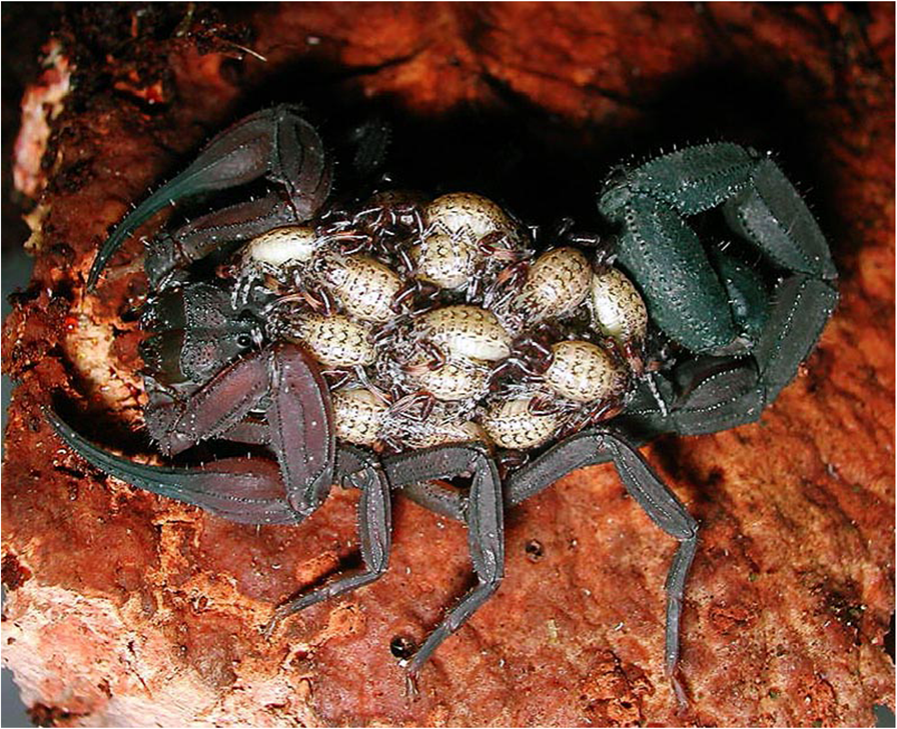
Female specimen of *Tityus ythieri* from Ecuador with an average litter size of first instar pro-juveniles (copyright by Eric Ythier, reproduced with permission)

**Fig. 13:**
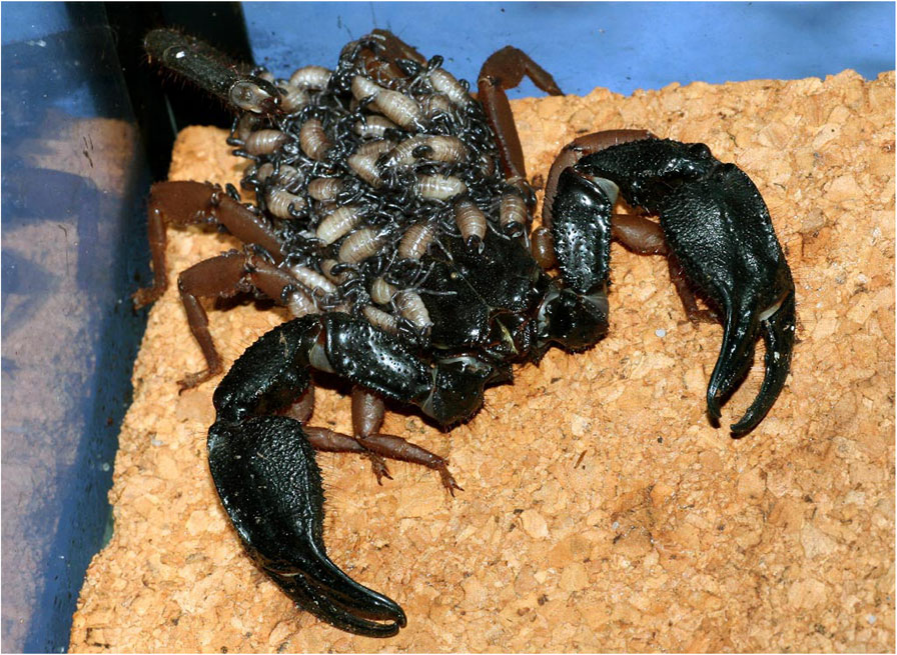
Female of *Chiromachus ochropus* from the Seychelles Islands with a huge litter size of first instar pro-juveniles

**Fig. 14:**
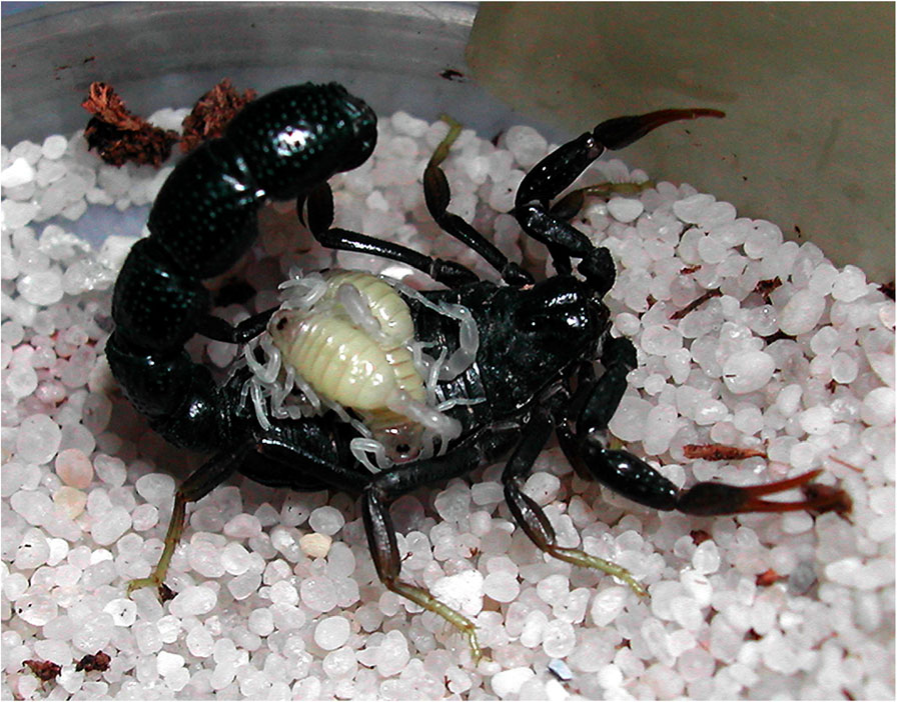
Female of *Orthochirus scrobiculosus* from Saudi Arabia with a reduced litter size of first instar pro-juveniles (copyright by Eric Ythier, reproduced with permission)

When the embryonic development is achieved, the birth process takes place. For details on the birth process refer to Polis and Sissom [8] and Lourenço [7]. After parturition all the young ascend and settle on the female’s back (Fig. 12). Litter size is variable, ranging from 3 to 4 to 105–110 young per brood; some Hormuridae such as *Chiromachus ochropus* from the Seychelles Islands may have broods surpassing 100 pro-juveniles [53] while several micro-buthoids present very small broods composed of sometimes only 2–3 pro-juveniles (Figs 13 and 14) [57]. Most other species show intermediate values ranging in general from 15 to 50 pro-juveniles, the range that also includes the brood sizes of most noxious species [7]. Brood size received little attention in previous publications and was first discussed by Francke [58]. This author attempted to explain the factors involved in the litter size of scorpions of the family Diplocentridae and concluded that litter size would be directly proportional to the size of the female and inversely proportional to the size of the young. Thus, the size of the mother and the size of the young were found to account for 81% of the variation in litter size between the species of this family. Polis and Sissom [8] briefly revisited this matter, but referred to Francke’s [58] results as being intuitive. The syntheses proposed by Polis and Sissom [8] and Lourenço [7, 59] only summarize the known data about litter size in scorpions, without further discussion of the factors that might be responsible for its variability. Subsequent to the publications by Polis and Sissom [8] and Lourenço [7], new data on litter size of several species of buthoid scorpions have become available and were summarized in a table by Lourenço [57] who gave special attention to micro-buthoid species [60], including members the families Buthidae Koch and Microcharmidae Lourenço.

Many, if not most, of these small species of buthids and microcharmids show a strongly reduced litter size, in most cases with numbers less than 10 and in others less than 7 juveniles. It is important to note that reduced broods have also been observed in large species of buthids, in which normal values range from 15 to 50. These weak numbers have frequently been reported as standard by several authors [8]; however, this interpretation is incorrect since this situation can be observed among females of any species arriving at the end of their reproductive life, when the number of follicles starts to decline [9]. These numbers should not be retained as standard average values.

In fact the number of juveniles in a litter is directly related not only to the scorpion lineage but also to the durations of embryonic and post-embryonic development, i.e. the number of molts necessary to reach adulthood. This leads to a number of conclusions: the size of litter size in a given species is independent of ecological factors, since species with similar brood sizes can be humicolous or live in rain forests while others inhabit arid environments and even deserts. In all cases, as already proposed by Francke [58], litter size is directly proportional to the size of the female and inversely proportional to the size of the young. The large body size of pro-juveniles at birth seems to be associated with a more ‘complete’ embryonic development. This factor may permit a strategy of post-embryonic development with a smaller number of instars. Morphometric growth values presented by pro-juveniles that belong to very small broods are higher than those observed generally in other buthoids. Finally, the sex ratio of most species within a given population is often close to 1:1, but some species have ratios of 3:1 or 4:1 in favor of females.

**Fig. 15:**
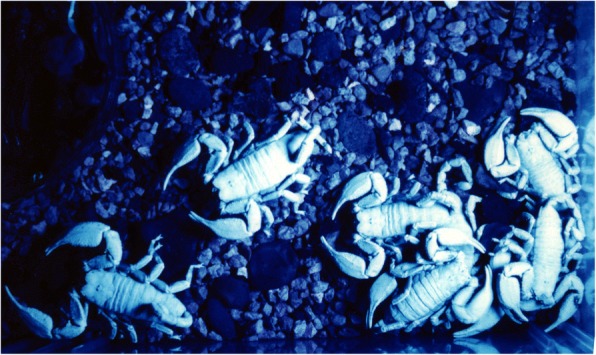
A group of the social species *Opisthacanthus cayaporum* (hormurid) under UV light

The young remain with their mother until their first molt and generally disperse afterwards. This period of contact between mother and juveniles may therefore last from 5 to 30 days, depending on the species. During this period, a rather sophisticated maternal behavior can be observed among all studied species [7, 59]. Some species can also present divers degrees of social behavior (Fig. 15). In these cases the young remain with the mother and other adults during all their lifetime. This type of behavior was notably presented by species of the families Hormuridae, Scorpionidae and Diplocentridae [12]. Remarkably, none of the species belonging to these lineages has an infamous reputation in relation to human incidents.

## Post-embryonic development

Post-embryonic development comprises the period after birth until the adult stage has been reached. It can be divided into two phases: pro-juvenile and juvenile. The pro-juvenile phase consists of a single instar that lasts from the moment of birth until the first molt. During this instar the young remain on their mother’s back (Figs. 12, 13 and 14). The first instar young cannot feed or sting. Their tarsi possess suckers instead of the ungues that appear only after the first molt. The duration of the pro-juvenile instar is variable, ranging in general from 5 to 25 days. The first molt takes place simultaneously among all the pro-juveniles in the litter. On average, it takes from 6 to 8 h. The juvenile phase begins after the first molt and comprises a variable number of instars that differ according to the species; a variation in the number of instars can also be observed within the same species [7–9, 59, 61]. The duration of a given instar is variable among juveniles of the same litter. However, in social species such as *Opisthacanthus cayaporum* (Fig. 15) most if not all the juveniles of the litter will molt within a short period of time, normally during the same night [62]. This behavior suggests a group effect.

**Fig. 16:**
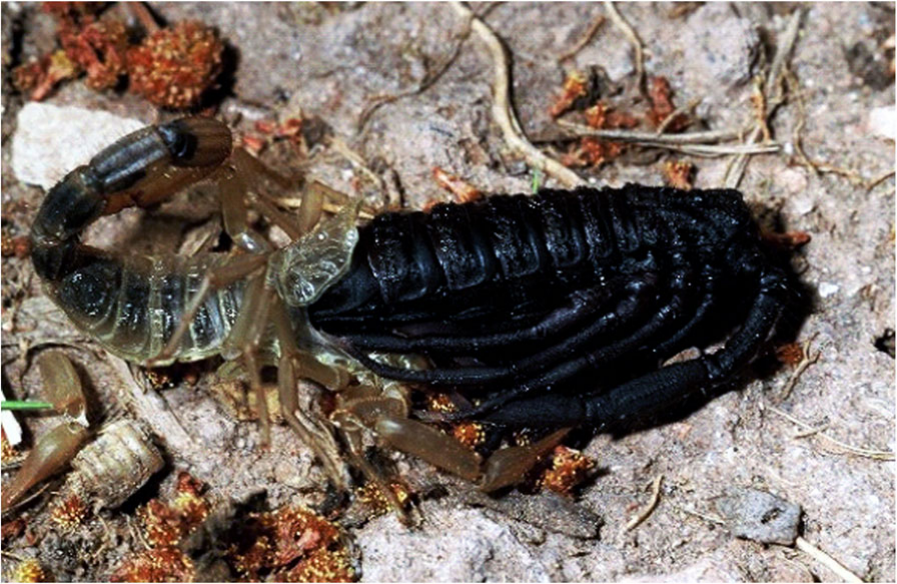
Molting process in the buthid scorpion *Hottentotta franzwerneri* from North Africa

**Fig. 17:**
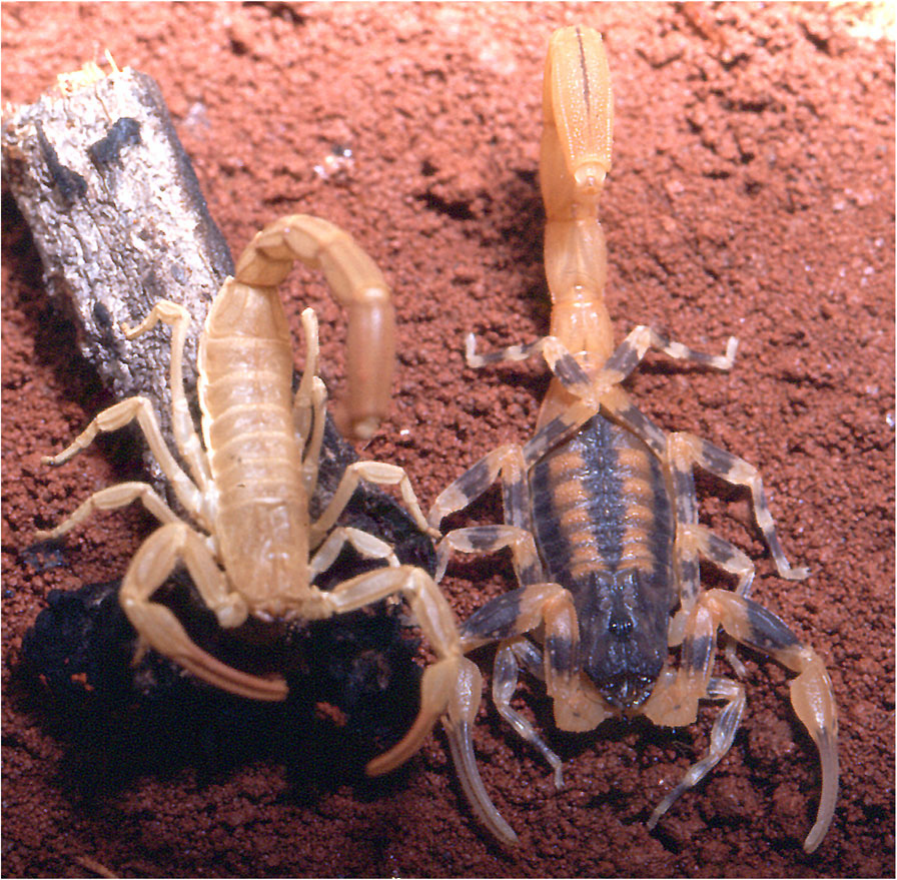
The exuviation process concluded for the buthid scorpion *Tityus fasciolatus* from Brazil

**Fig. 18:**
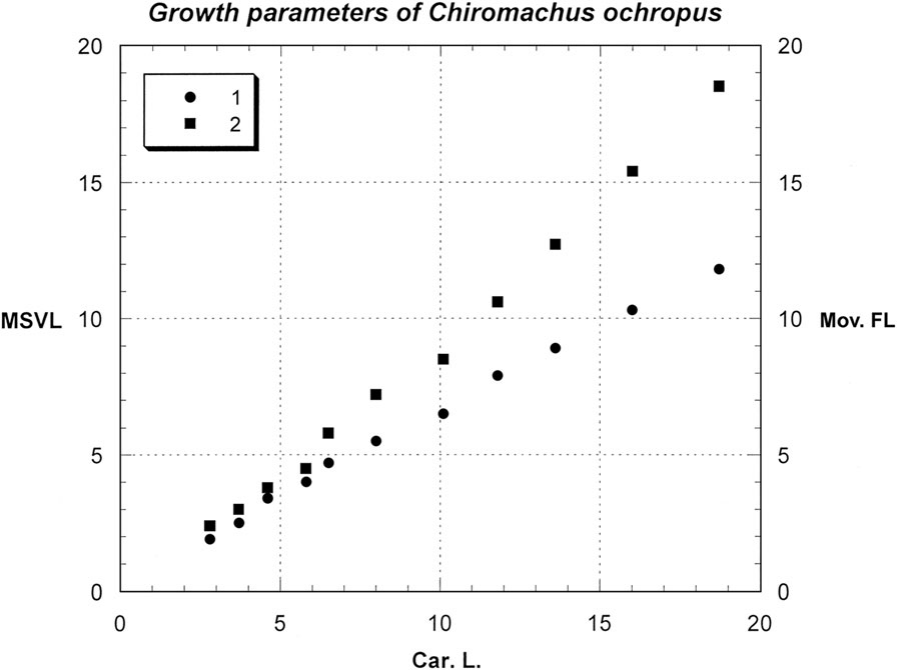
Graph showing the growth parameters in the scorpion *Chiromachus ochropus.* Values are calculated from carapace length (Car. L.), metasomal segment V length (MSVL) and movable finger length (Mov. FL). The number of instars reaches 12 in this species

Before molting, scorpions become reclusive and inactive until the cuticle has been shed, possibly by blood pressure (Figs. 16, 17). The cuticle ruptures at the sides and front margin of the carapace, while the chelicerae, pedipalps and legs are withdrawn from the exuviae. The body emerges slowly during short periods of vigorous movement that alternate with long periods of relaxation. The process usually takes place in well-hidden places or during the night. It lasts from 10 to 14 h. Immediately after molting the scorpion cuticle is not fluorescent under UV light, and does not become so until the new cuticle hardens. The exuviae are, however, fluorescent. The duration of the different instars is variable and depends on the ambient temperature, humidity and food. The total number of instars observed may vary strongly, from 4 in some micro-buthoids [57] to 12 in the *Chiromachus ochropus* (Fig. 18), a hormurid endemic to the Seychelles Islands [53].

In several species, males and females can be distinguished only after the last molt when sexual dimorphism becomes visible. This is the case in several species from the buthid or chactid genera, such as *Tityus*, *Centruroides*, *Babycurus*, *Brotheas* and *Broteochactas*, etc. In other buthid or ischnurid species, such as those belonging to the genera *Grosphus* and *Opisthacanthus*, sexual dimorphism is apparent from birth and the sexes can easily be recognized after the first juvenile instar (instar 2).

## Lifespan

The lifespan of scorpions is variable and may be extraordinarily long, ranging from 4 to 25 years [7, 8]. We still know nothing about the life histories of most small scorpion species, so new data may reveal more short-lived species.

## Parthenogenesis

Parthenogenesis (from the Greek παρθενος *parthenos* = ‘virgin’ + γενεσις *genesis* = ‘birth’) is a form of reproduction in which the ovum develops without fertilization. This phenomenon in scorpions was previously outlined in previous papers [5, 63]; however, since this last synthesis some more data became available and are herein summarized.

**Fig. 19:**
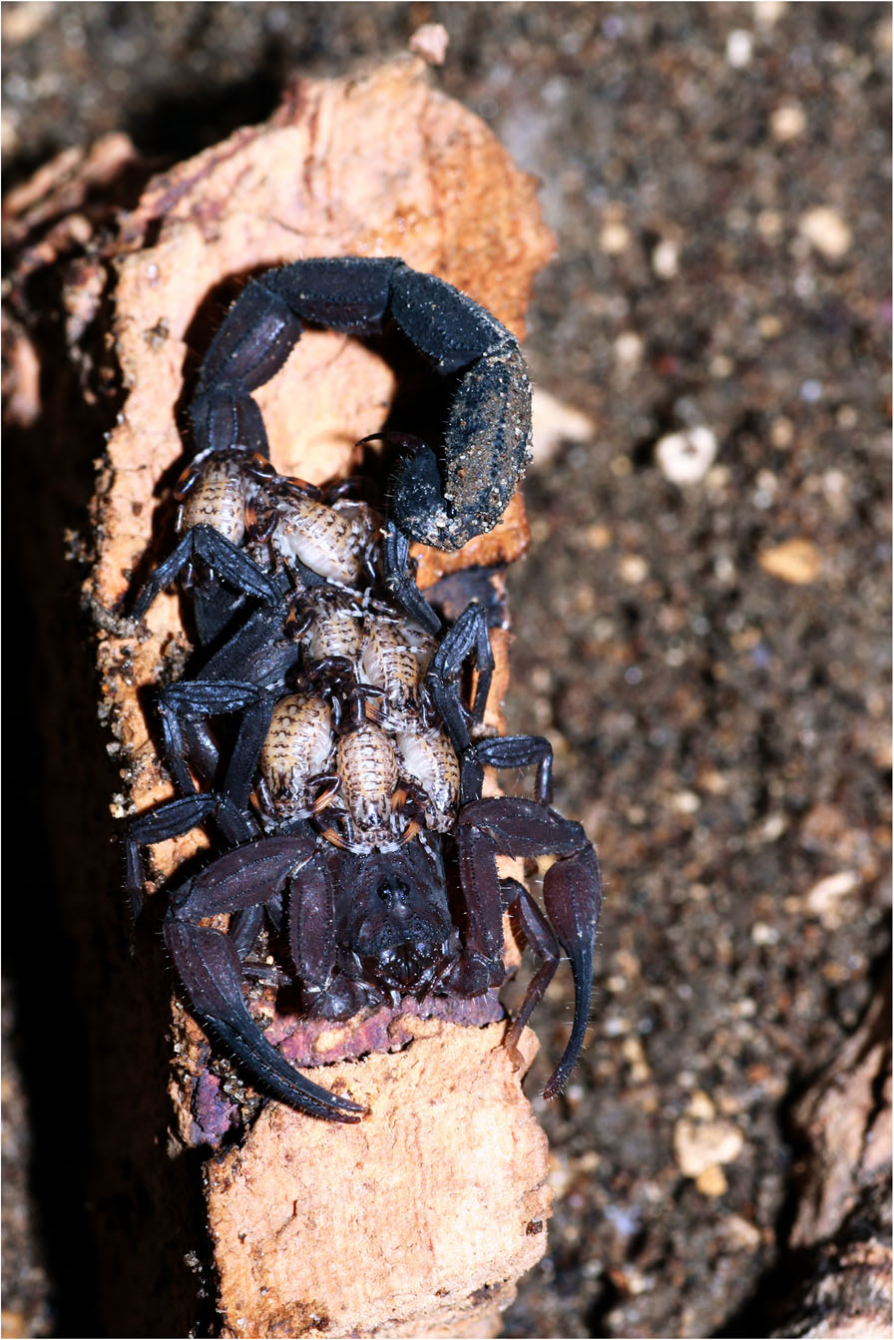
Parthenogenetic female of *Tityus neblina* from the Tepui Neblina (Brazil-Venezuela) with first instar pro-juveniles

Thelytokous parthenogenesis (with all-female broods) is the general trend observed among scorpions [63]. The most classical example being the noxious Brazilian species *Tityus serrulatus*. However, two known exceptions are the species *Tityus metuendus* Pocock from the western Amazon and *Tityus neblina* Lourenço from the Tepui ‘Pico da Neblina’, located between Brazil and Venezuela. As to the first, a unique case of arrhenotokous (all-male broods) was confirmed [64, 65], while in the second a first case of deutherotokous (male and female brood) was observed (Fig. 19) [11, 48].

Historically, this very peculiar phenomenon was first described in the Brazilian buthid *Tityus serrulatus* [25]. Most new findings about this reproduction process in scorpions were confirmed after the 1990s and 2000s, in contrast with the suggestion of Polis and Sissom [8] to whom this phenomenon would be rare.

Among more than 2200 species of scorpions distributed throughout the world, about 15 are known or at least have been suggested to be parthenogenetic [5, 63, 64, 66]. After the original report by Matthiesen [25] on *Tityus serrulatus*, several other species proved to be parthenogenetic. The majority of the reports concern species of the family Buthidae that belong to the genera *Tityus*, *Hottentota*, *Ananteris*, *Lychas* and *Pseudolychas*. Other reports are also known for non-buthid species that belong to the genera *Liochelis* (Hormuridae) and *Serradigitus* (Vaejovidae).

The parthenogenetic pattern observed in scorpions corresponds in all cases to the model defined by Vandel [67] as ‘geographic parthenogenesis’, and can be tentatively explained in terms of the life-history strategies of the populations. However, very few studies are available on the comparative dynamics of parthenogenetic vs. sexual populations. The single exception is the comparative study carried out by Lourenço et al. [68] on the Colombian populations of *Tityus columbianus*. The authors reported that most ecological parameters have been compared between parthenogenetic and sexual aspects, and demonstrated that the sexual females were significantly larger and had significantly greater relative litter mass than the parthenogenetic ones [68].

## Conclusions

Reproductive strategies are intrinsically associated with the population dynamics of each scorpion species. Consequently, species presenting short life cycles, capacity to store spermatozoa and the potential to reproduce asexually (parthenogenesis) will be preferentially selected in newly created environments generally disturbed by human action. The longer life cycles seem to predominate in older lineages with narrow-ranged species, but variability is also observed in these groups, and may have been the key for the adaptation of so many scorpions in a diversity of habitats on all continents.

When these reproductive parameters are associated with strongly opportunistic species capable of colonizing disturbed and unpredictable environments, one can observe populations ‘explosions’, since in these cases regulations are not of the density-dependent type, but rather of the catastrophic type [59, 69]. This basic model seems to be perfectly applicable to several infamous species of *Tityus* in South America, *Centruroides* in North America and *Androctonus* in North Africa [2–4].
